# Supplementary material for: Content-rich biological network constructed by mining PubMed abstracts
Source: BMC Bioinformatics. 2004 Oct 8;5:147. doi: 10.1186/1471-2105-5-147 (PMC528731; doi:10.1186/1471-2105-5-147)
Supplement: Additional File 5 — The original Chilibot query results of the term "long-term potentiation (LTP)" and 22 other terms, limiting the latest references analyzed to the years 1990, 1995, 2000, and 2004. [file 1471-2105-5-147-S5.bz2 › chilibotAdditionalFile5/ltp2004/html/ERK_ZIF268.html]

 


 **ERK** and **ZIF268** 
  
Found 9 abstracts in PubMed,  **9 abstracts were retrieved and analyzed**.  


---

 Search Google  |
 PDF files only 
|  EDU domain only 

---

**Interactive relationship** (e.g. stimulation, inhibition, etc)

- Furthermore, THC induced phosphorylation of the transcription factor Elk 1, and up regulation of  **zif268**  mRNA expression are blocked by SL327, a specific inhibitor of MAPK  **ERK**  kinase MEK, the upstream kinase of  **ERK** , as well as SCH 23390.  Ref: 11553284 Eur J Neurosci, 2001

- :-)

  **Parallel relationship** (e.g. studied together, co-existance, homology, etc.)

  - In this study we examined the contribution of MAPK1 and 2 also known as extracellular signal regulated kinases  **ERK**  1 and 2 to the induction of  **zif268**  mRNA in PC12D cells by using two methods to block the activation of these kinases.  Ref: 10050043 J Biochem (Tokyo), 1999
  - We show that pharmacological blockade of MAPK  **ERK**  phosphorylation, functional inactivation of CREB in an inducible transgenic mouse and inactivation of  **zif268**  in a mutant mouse result in a similar deficit in long term recognition memory.  Ref: 12740127 Philos Trans R Soc Lond B Biol Sci, 2003
  - Following a similar time course, the two downstream transcriptional targets of MAPK  **ERK** , cAMP response element binding protein CREB and the ternary complex factor Elk 1, a key transcriptional regulator of serum response element SRE driven gene expression, were hyperphosphorylated and the immediate early gene  **zif268**  was upregulated.  Ref: 10844026 J Neurosci, 2000
  - Here, we briefly review experiments that have shown MAPK  **ERK** , cAMP response element binding protein CREB and the immediate early gene IEG  **zif268**  are essential components of a signalling cascade required for the expression of late phase LTP and of certain forms of long term memory.  Ref: 12740127 Philos Trans R Soc Lond B Biol Sci, 2003
